# Laser Micromachining of Liquid Metal Patterns for Stretchable Electronic Circuits

**DOI:** 10.1002/admt.202501838

**Published:** 2025-12-12

**Authors:** Merjen Palvanova, Patrick McManigal, Grace Fredrickson, Eric J. Markvicka

**Affiliations:** 1 Department of Mechanical & Materials Engineering, Smart Materials and Robotics Laboratory, University of Nebraska-Lincoln, Lincoln, USA; 2 School of Computing, College of Engineering, University of Nebraska-Lincoln, Lincoln, USA; 3 School of Computing, College of Engineering, Smart Materials and Robotics Laboratory, University of Nebraska-Lincoln, Lincoln, USA

**Keywords:** biomonitoring, liquid metal, laser micromachining, stretchable electronics

## Abstract

Stretchable electronic circuits are crucial for wearable computing, soft robotics, and bio-integrated devices. Gallium-based liquid metal (LM) conductors offer exceptional electrical and mechanical properties, yet they are difficult to pattern due to their high surface tension and weak adhesion to several surfaces. Here, we introduce a rapid (<3 h), cost-effective (~;$15/device) fabrication approach that combines UV-laser micromachining with a facile copper foil wetting layer to enable high-resolution patterning of LM circuits on diverse substrates, including elastomers, fabrics, and adhesives. This cleanroom-free process achieves high resolution (60 μm) and improved electrical interfacing through hydrochloric acid vapor treatment. The resulting circuits maintain functionality under high strain (>100%) and cyclic loading. To demonstrate the fabrication approach, a wearable pulse oximeter is created for real-time monitoring of heart rate. This scalable and maskless fabrication approach broadens the accessibility of stretchable LM-based electronics by reducing production costs and enabling rapid prototyping for next-generation wearable and soft robotic systems.

## 1 ∣ Introduction

The future of wearable computing, soft robotics, and health monitoring will increasingly rely on soft and deformable electronic circuits that closely mimic the mechanical properties of natural biological systems [1, 2]. This emerging field of stretchable electronics is driven by two prevailing approaches, each offering unique advantages and challenges. The first approach involves integrating high-performance electronic components with thin, bendable, yet inextensible films [3-8]. This method relies on patterning intrinsically rigid materials (elastic moduli ≥ 1 GPa) into deterministic architectures using cleanroom fabrication techniques for thin-film deposition and patterning of conductive and insulating materials. These materials are patterned into structures, such as serpentine or fractal patterns, to enable stretchable functionality while maintaining the high performance of traditional electronics. The second approach utilizes intrinsically soft and conductive polymers and composites. This method involves doping insulating polymers (e.g., polydimethylsiloxane, polyurethane, polyacrylate, or fluoropolymer) with conductive fillers such as rigid metallic nano-/microparticles [9-12], carbon allotropes [13-16], and other conductive groups [17, 18] to form percolating networks. While these composites are promising, they often face challenges in terms of mechanical properties and electrical conductivity. Despite some of these limitations, the composite materials have shown significant potential in low-load/moderate-strain applications.

Liquid metal (LM)-based conductors represent a promising alternative for creating stretchable electronics. Gallium-based alloys like eutectic Ga–In (EGaIn; 75% Ga and 25% In, by weight) and Ga–In–Sn (Galinstan; 68% Ga, 22% In, and 10% Sn, by weight) are particularly promising due to their low melting point (−19°C for Galinstan, 15°C for EGaIn), high electrical conductivity (3.4 × 10^6^ S × m^−1^), negligible toxicity, and minimal vapor pressure [19]. Their low viscosity (2 mPa × s) and fluidic nature enable seamless integration into soft elastomers, surpassing the geometric constraints of deterministic architectures and outperforming conductive polymers in both elasticity and conductivity. However, the high surface tension of Ga-based LMs (∼624 mN · m^−1^) [19], poses significant challenges in wetting most surfaces, often causing spontaneous droplet formation to minimize surface energy [20]. To overcome these challenges, advanced patterning methods have been introduced for creating microfluidic channels of LM, such as direct ink writing [21-29], stencil lithography [30-38], transfer printing [39, 40], selective wetting/intermetallic alloying [41-48], and laser micromachining [44, 47, 49-51]. However, the electrically insulating, self-passivating gallium oxide (Ga_2_O_3_) layer poses challenges for microelectronic circuit integration. To improve the interface between microelectronic components and LM microfluidic channels, hydrochloric acid (HCl) vapor treatment can be used to remove the Ga-oxide layer, thereby reducing interfacial contact resistance [43]. However, the HCl vapor treatment requires a wetting layer to maintain the circuit pattern when the Ga-oxide layer is removed. Previously, researchers have used a copper wetting layer that is sputter deposited onto the elastomer substrate with an intermediate chromium adhesion layer under high vacuum (10^−9^ torr) within a cleanroom facility [42-44]. The high vacuum deposition environment ultimately restricts the compatible substrate options due to outgassing during pump down, which preclude the use of highly extensible elastomers commonly used in stretchable electronics, such as EcoFlex or Dragon Skin (Smooth-On). The low outgassing tolerance, high cost, and lengthy processing time highlight the need for alternative fabrication strategies that don’t rely on cleanroom-based fabrication processes and support a broader range of substrate materials.

Here, we introduce a novel method for rapidly producing stretchable electronic circuits by combining UV-laser micromachining with a simple copper-based wetting layer. Unlike existing LM patterning techniques, our approach eliminates the need for specialized cleanroom facilities for sputter deposition, which simplifies the fabrication process and enables compatibility with a wider range of substrate materials. Electrical traces are created by bonding a thin copper film to the desired substrate, which is then coated in LM. High-resolution circuit features are patterned utilizing UV-laser micromachining, allowing fabrication without photomasks and enabling seamless integration with existing PCB workflows.

**FIGURE 1 ∣:**
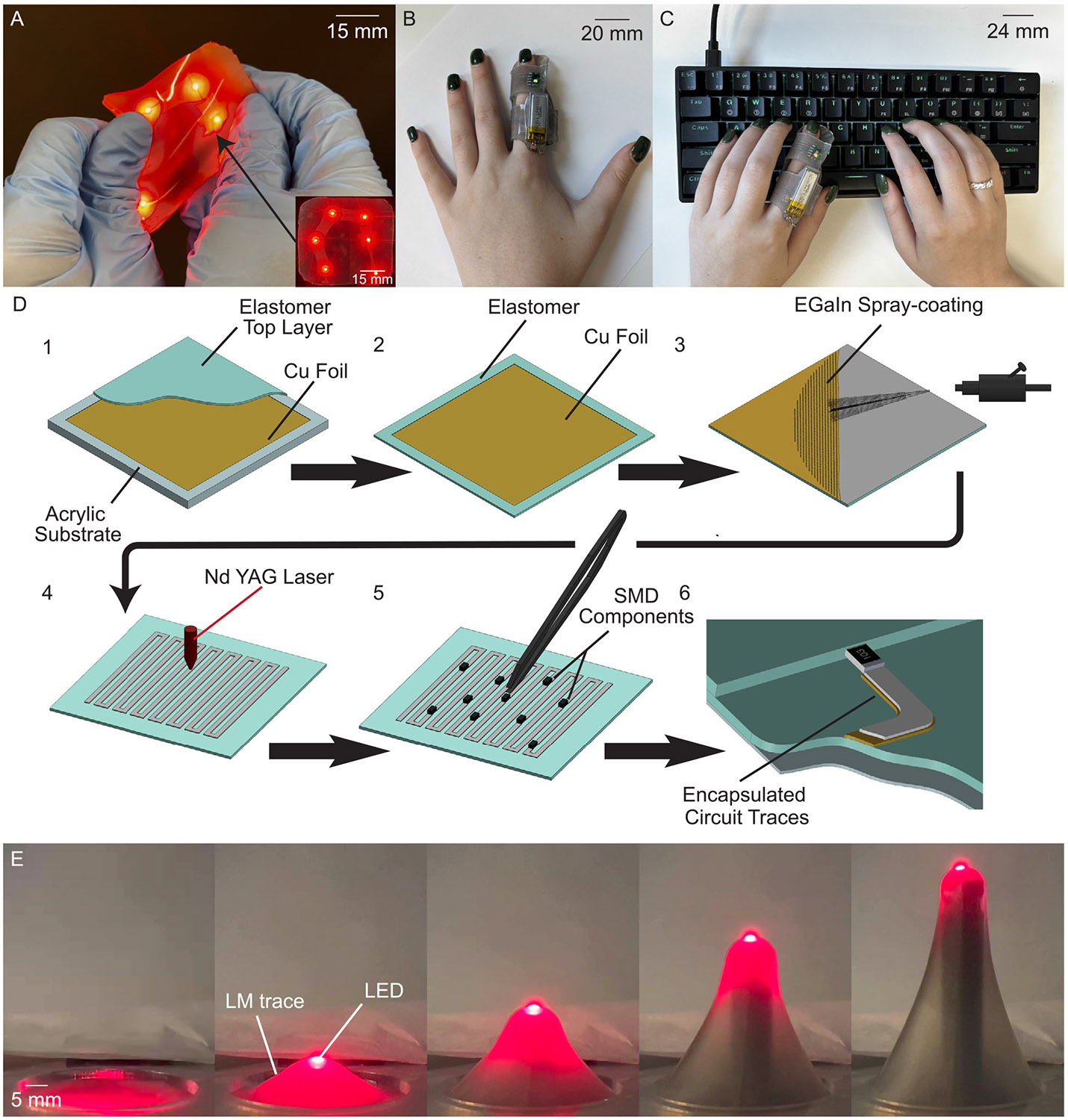
Fabrication of LM-based stretchable electronics. (A) Photograph of an LED circuit that is able to sustain large deformation during operation. The inset shows the non-stretched circuit. (B,C) Photograph of a stretchable pulse oximeter circuit wrapped around the finger for measuring heart rate. (D) Schematic illustration of the fabrication process: (1) Cu foil is placed onto an acrylic substrate, spin-coated with elastomer, and cured in an oven. (2) To expose the Cu film, the substrate is flipped over. (3) The Cu surface is spray-coated with 2 passes of EGaIn. (4) Circuit traces are patterned using UV laser micromachining. (5) HCl-EGaIn treated surface mount components are placed onto the circuit traces. (6) The circuit is sealed with a elastomer. (E) Video frames of the continued functionality of a LED during extreme deformation of a stretchable LM circuit during stretching and bending.

The approach is compatible with challenging substrates, such as those prone to outgassing, including as Ecoflex and Dragon Skin (Smooth-On). In addition, the process eliminates the dependence on high vacuum, cleanroom-based thin film deposition techniques, enabling a rapid (< 3 h), cost-effective (∼ $15 per 80 cm^2^ device, Table S1), and a versatile fabrication process. Importantly, devices with a wide range of designs and pattern densities can be created within the same time frame, as the change in laser processing time is negligible compared to the curing of the elastomer layers and other processing steps. Additional time may be required when components have temperature limitations. For comparison in terms of cost, a standard rigid 2-layer printed circuit board costs $5 per 6.5 cm^2^ without components (OSH Park). Surface-mount electrical components wetted with EGaIn can be directly assembled onto the LM traces. HCl is used to remove the Ga-oxide skin, reducing the contact resistance between the LM conductors and microelectronic components. Importantly, the copper film serves as a LM wetting layer, allowing the LM to maintain the patterned shape even after the Ga-oxide skin is removed. As shown in Figure 1A-C, we demonstrate the capabilities of this approach by fabricating a soft, stretchable circuit that adheres to the skin and integrates onboard processing, power, and wireless communication for remote biomonitoring. The resulting LM-based circuits exhibit robust performance even under substantial deformation, as shown in Figure 1E and Videos S1 and S2.

## 2 ∣ Results and Discussion

### 2.1 ∣ Fabrication Methodology

Fabrication of the LM-based stretchable electronic circuits follows four main steps: (1) circuit design, (2) LM circuit fabrication, (3) electronics interfacing, and (4) elastomer encapsulation, as illustrated in Figure 1D and detailed in the Experimental Section and Video S3. The process begins with circuit design using industry-standard PCB software (e.g., Altium Designer). For LM circuit fabrication, a bilayer structure is created by spin-coating polydimethylsiloxane (PDMS; Sylgard 184; Dow) onto copper foil mounted on an acrylic substrate, followed by curing at 80°C for 20 min (Figure 1D step 1). After removing and inverting this structure, EGaIn is spray-coated onto the exposed copper surface (Figure 1D steps 2 and 3). The high impact energy of the atomized EGaIn droplets ruptures the Ga-oxide layer upon impact, allowing the LM to wet the copper surface and form a uniform, high-quality EGaIn film [52]. UV-laser micromachining then precisely patterns the Cu-LM circuit traces (Figure 1D step 4). UV-laser micromachining ranges from 10 to 40 min, depending on the size of the circuit pattern. After patterning, the thickness of the deposited Cu-LM layer was measured (Figure S1E). The EGaIn layer has a thickness of 85 ± 5 μm. For electronics interfacing, the surface-mount components are treated with HCl, wet with LM, and directly placed onto the LM traces, removing the gallium oxide skin and enhancing the electrical contact with the LM traces (Figure 1D step 5; Video S4). Notably, the copper foil wetting layer allows the LM to maintain the shape of the circuit pattern during the placement of the HCl treated components. Finally, an elastomer prepolymer is poured over the circuit and cured at 60°C for 40 min to encapsulate the device (Figure 1D step 6). As demonstrated in Figure 1E and Videos S1 and S2, an LM circuit with a red LED remains operational under extreme deformation.

### 2.2 ∣ Characterization of Liquid Metal Patterning

**FIGURE 2 ∣:**
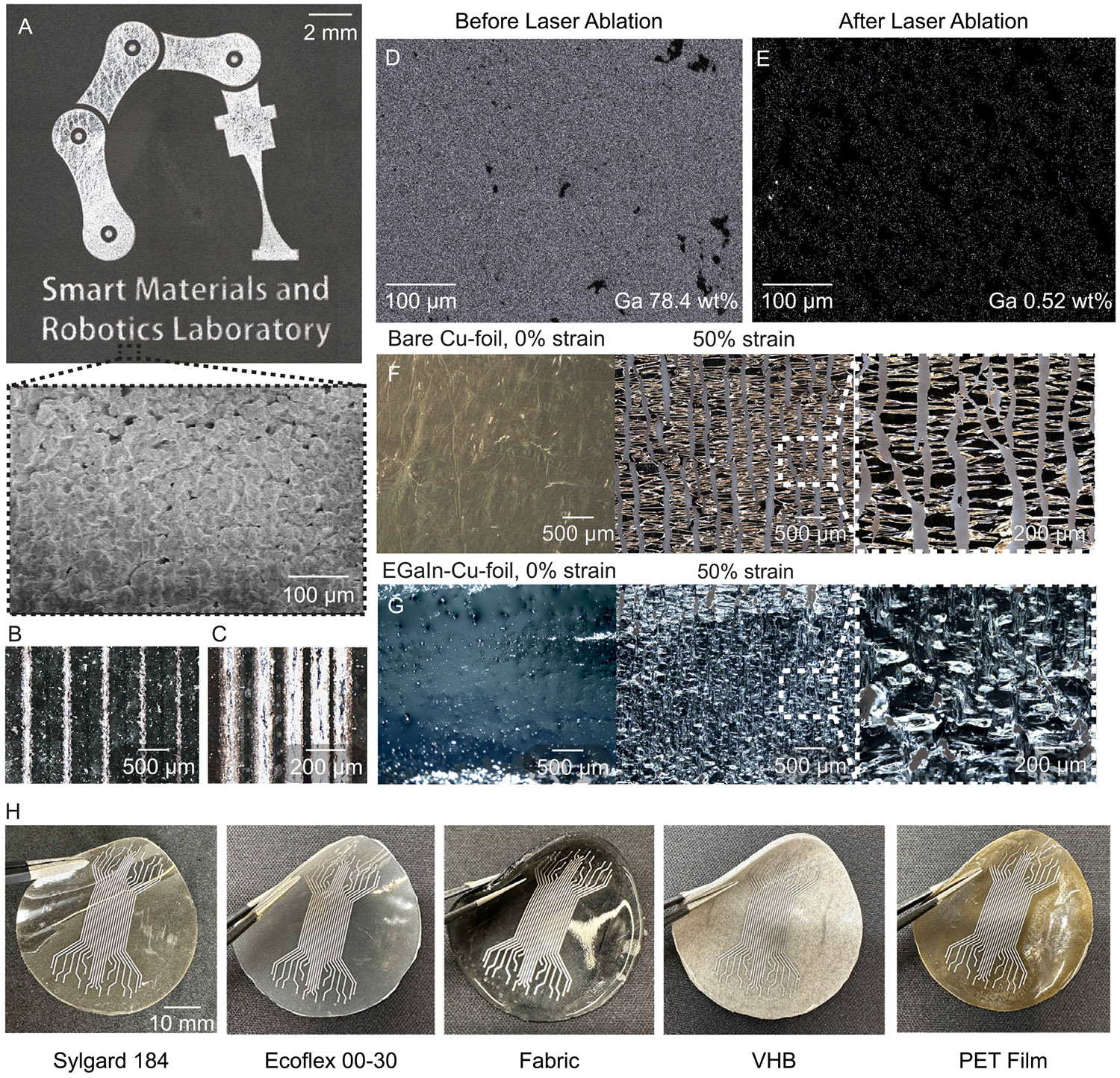
UV laser patterned LM features on various substrate materials. (A) Cu-LM features are patterned on a PDMS substrate. (inset) The SEM image shows the surface is also structured as a result of laser ablation. The dimensions of patterned traces can be as small as 20 μm for both trace (B) width and (C) spacing. The EDX image of elemental distribution of Ga (D) before and (E) after UV laser ablation. (E) Negligible residual Ga remains after UV laser patterning. (F) Bare Cu foil ruptures and loses conductivity when strained to 50%. (G) In contrast, when coated with LM, the LM bridges the Cu islands and ensures continuous conductivity. (H) LM traces can be patterned on a wide range of stretchable materials commonly used in stretchable electronics, including silicone elastomers, elastic fabric adhesives, pressure-sensitive adhesives, and plastic films.

The rapid fabrication of soft and stretchable LM circuits was accomplished through a combination of a Cu-based wetting layer and UV-laser micromachining. As shown in Figure 2A, the Cu-LM bilayer on PDMS can be patterned into features with high resolution. The scanning electron microscopy reveals that laser ablation not only removes the Cu-LM film but also textures the elastomer surface (Figure 2A inset). As shown in Figure 2B,C, the minimum trace width and spacing were 60 μm, which is significantly finer than the 150 μm standard in conventional PCB manufacturing. Finer minimum trace width and spacing (20 μm) was possible. However, minor defects in the Cu-LM layer, arising from imperfections in the copper foil or impurities in the EGaIn, can cause a loss of continuity. After patterning, the Cu–LM layer thickness was characterized (Figure S1E), the EGaIn layer had a uniform thickness of 85 ± 5 μm across the patterned regions. The effectiveness of laser micromachining was further assessed by energy dispersive X-ray spectroscopy (EDX) to compare the Ga content on the elastomer surface before and after laser ablation (Figure 2D,E). Initially, the coated surface exhibits Ga content similar to bulk EGaIn (78.4 wt.%), whereas after ablation, only a negligible amount remains (0.52 wt.%). Figure 2F,G shows micrographs of the Cu foil before and after LM coating, respectively. When stretched to 50% strain, the uncoated Cu foil fractures, while the EGaIn coated on the Cu surface fills the cracks and voids, preserving electrical conductivity under strain. The magnified photographs shown in the inset of Figure 2F,G further illustrate the Cu-LM architecture and the LM’s ability to maintain electrical conductivity by bridging the cracks within the Cu foil.

To demonstrate the versatility of this fabrication method, Cu-LM traces were patterned onto various commonly used materials, including silicone elastomers (Sylgard 184, Dow; Ecoflex 00-30, Smooth-On), a soft and elastic polyurethane fabric adhesive (2mil 3412, Bemis), a pressure-sensitive adhesive (VHB 4910, 3M), and plastic (polyethylene terephthalate). As shown in Figure 2H, high-resolution LM traces can be patterned on these materials or attached using an adhesive film. This demonstrates the broad applicability of the proposed patterning method across diverse and challenging substrates, regardless of their outgassing properties.

### 2.3 ∣ Electromechanical Characterization and Fatigue Performance

**FIGURE 3 ∣:**
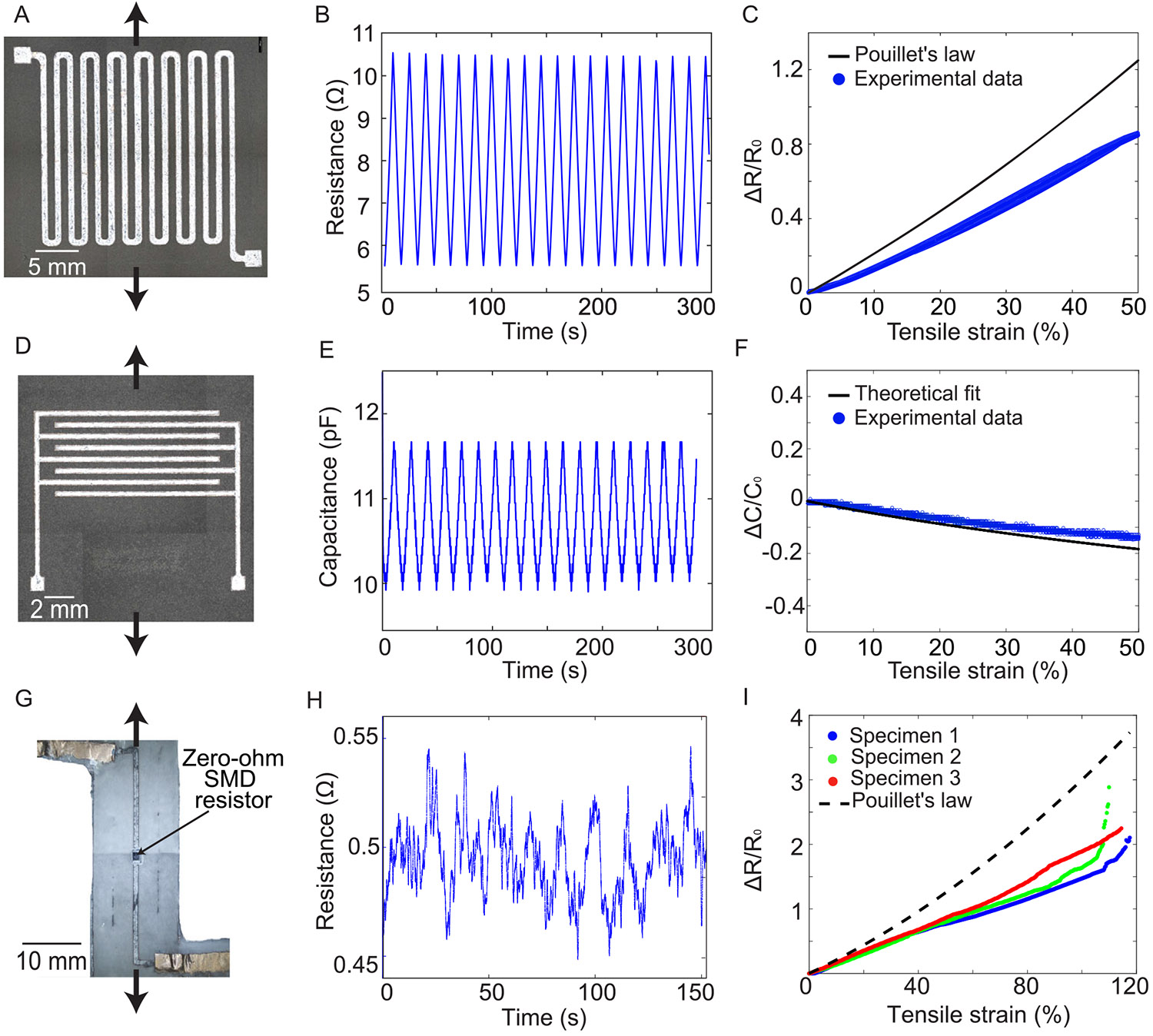
Electromechancial response of laser patterned LM traces. (A) Photograph of a serpentine stain sensor. (B) Experimental measurements of electrical resistance during 20 cycles up to 50% tensile strain plotted vs. time. (C) Normalized change in resistance vs. strain in comparison to Pouillet’s Law (black curve). (D) Photograph of a capacitive sensor with interdigitated electrodes. (E) Experimental measurements of capacitance during 20 cycles up to 50% tensile strain plotted vs. time. (F) Normalized change in capacitance vs. strain in comparison to electrostatic field theory (black curve). (G) Photograph of a sample with zero-ohm surface mount resistor. (H) Experimental measurements of electrical resistance during 10 cycles up to 50% tensile strain are plotted vs. time. (I) Normalized change in resistance vs. tensile strain to electrical failure and compared to Pouillet’s Law (black curve).

The electromechanical performance of the UV laser patterned LM traces was examined using various circuit designs, as shown in Figure 3. Unless otherwise noted, all circuit designs were patterned on and encapsulated using Ecoflex 00–30 (Smooth-On). First, a strain sensor was created using a serpentine trace that was 21 mm long and 0.76 mm wide (Figure 3A). The sensor was strained for 1000 cycles up to 50% strain (Figure 3B). When plotting these data as a function of strain, the loading and unloading curves are indistinguishable, showing that the sensor has a very low hysteresis upon cyclic loading (Figure 3C). The normalized change in resistance as a function of strain was compared to Pouillet’s Law (black curve): $\Delta R∕R_{0}={\left(\varepsilon +1\right)}^{2}-1$, where $R_{0}$ = 5.72 Ω. The strain sensor response is consistently lower than the theoretically predicted increase in resistance, suggesting that the Cu-LM traces are well-suited for PCB applications. The change in resistance could be further minimized by introducing wrinkling of the Cu foil or by optimizing the conductor geometry with wavy or serpentine patterns.

A planar capacitor with interdigitated electrodes was then fabricated with 0.38 mm trace width and spacing and 11 mm overlap (Figure 3D). The sensor was strained for 20 loading cycles up to 50% strain (Figure 3E). Similar to the resistive strain sensor, the capacitive sensor exhibited minimal hysteresis under cyclic loading (Figure 3F). The normalized change in capacitance as a function of strain was compared to electrostatic field theory (black curve): $\Delta C∕C_{0}=(1∕\sqrt{\varepsilon +1})-1$ for uniaxial strain perpendicular to the interdigitated electrodes [53, 54]. The measured increase in capacitance was similar to the theoretical fit, demonstrating adequate stability during cyclic loading.

A surface mount zero-ohm resistor was then integrated with the patterned LM traces and oriented along the loading direction (Figure 3G). The sample was strained for 20 cycles up to 50% strain (Figure 3H). A minimal change in absolute resistance was observed as a function of cyclic loading. Three samples were then strained until electrical failure. The normalized change in resistance as a function of strain was compared to Pouillet’s Law, where *R*_0_ = 0.60 Ω. As shown in Figure 3I, three samples were able to withstand strains up to 110% before experiencing electrical failure. All exhibited a negligible increase in absolute resistance (< 0.25 Ω) and a slightly lower resistance change than predicted by Pouillet’s Law. Failure occurred due to delamination of the encapsulating elastomer from the copper leads and surface mount component, which broke the connection between the component and LM trace. Robust circuit performance at a higher strain limit could be achieved by increasing the adhesion strength between the encapsulating elastomer and the surface mount component.

### 2.4 ∣ Wearable Pulse Oximeter

**FIGURE 4 ∣:**
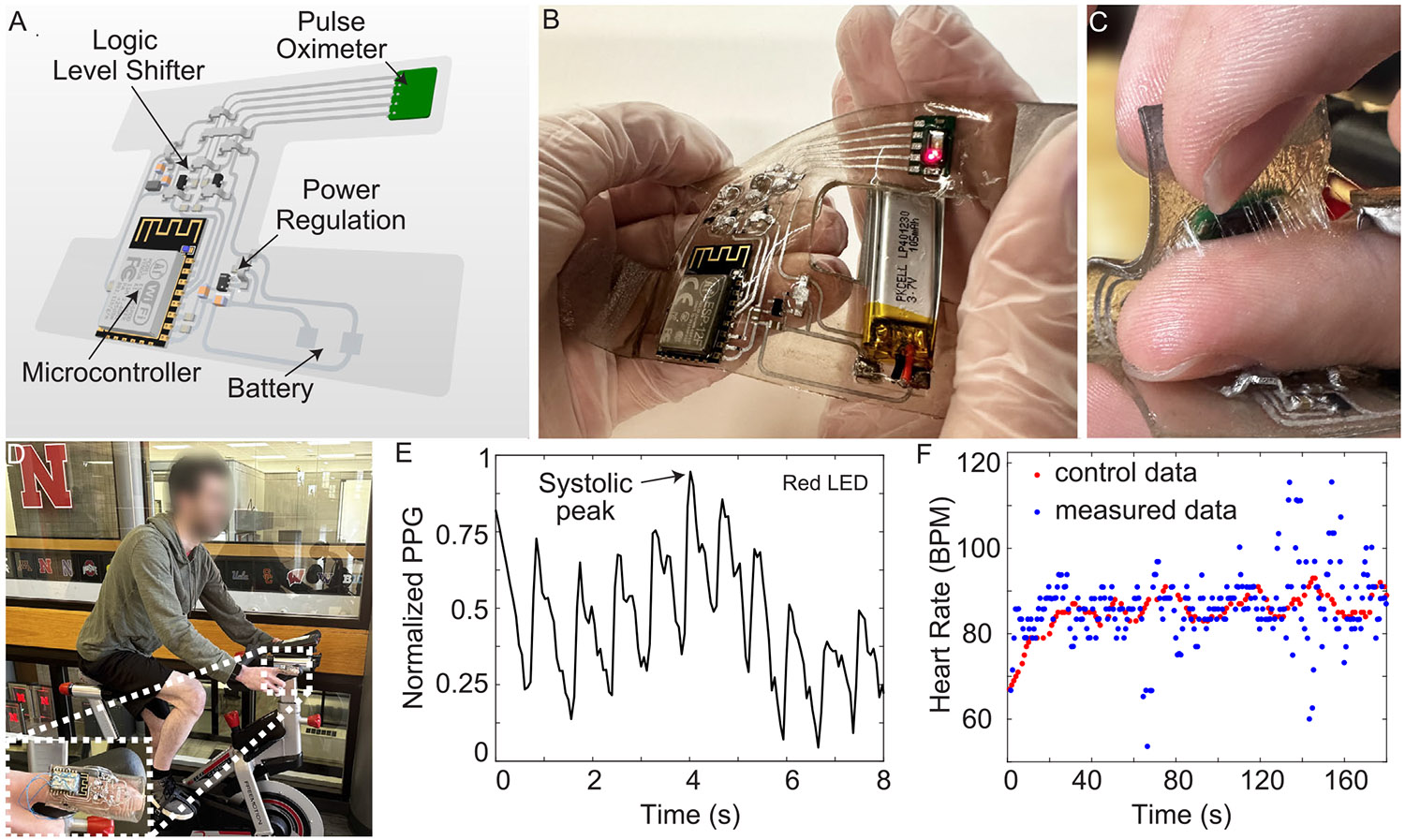
Soft matter pulse oximeter circuit. (A) Schematic illustration of the circuit with functional blocks indicated. (B) Device remains functional under deformation. (C) The elastomer encapsulation layer was modified by adding PEIE to increase the adhesion strength. (D) The wearable pulse oximeter mounted on the index finger of a cyclist on a stationary bicycle. (E) The waveform recorded from the pulse oximeter showing multiple cardiac cycles. (F) The measured heart rate as a function of time is compared to a commercial fingertip pulse oximeter.

The fabrication methodology was then used to create a fully untethered, soft and stretchable electronic circuit. Here, we created a wearable pulse oximeter that is capable of noninvasive continuous monitoring of heart rate (Figure 4). As illustrated in Figure 4A, the circuit contains an integrated pulse oximeter with red and IR LEDs, photodetector, and analog front end (MAX30101; Analog Devices Inc.); a low-cost Wi-Fi microcontroller with data interface and integrated antenna (ESP8266, Espressif Systems); logic level shifter; power regulation; and rechargeable battery (3.7 V, 400 mAh). Detailed fabrication of the device is shown in Figure S2. The soft device is capable of bending and stretching (Figure 4B) and consists of a combination of soft materials and rigid surface mount components that are sequentially assembled through a bottom-up process shown in Figure S2. To attach the wearable device to the human body, the adhesion strength of the PMDS elastomer was tuned by adding ethoxylated polyethylenimine (PEIE). The amine-based PEIE reacts with the curing agent, resulting in unreacted precursors distributed throughout the matrix, thereby increasing the adhesion [55]. The circuit was fabricated as previously described and then subsequently coated with PDMS containing 0.125 wt.% PEIE to form an adhesive layer (Figure 4C). The soft and stretchable pulse oximeter was created in approximately 11 hours, and the total cost was ∼$15/device plus the cost of the microelectronic components (Table S1). For comparison, a standard rigid 2-layer printed circuit board costs $5 per 6.5 cm^2^ without components (OSH Park). The total fabrication time for this circuit was increased because of the thermal limitations of the rechargeable battery, which had to be cured at a lower temperature.

The functionality of the wearable pulse oximeter was evaluated during cycling on a stationary bicycle (Figure 4D). Data was collected at a frequency of 16.7 Hz and uploaded to a cloud database in real time. A peak-finding algorithm identified Systolic peaks from the light backscattered or reflected by the red LED. A subsection of the recorded data is presented in Figure 4E. Heart rate was calculated by measuring the time intervals between Systolic peaks and averaging over a 3 second window. As shown in Figure 4F, the calculated heart rate closely aligns with measurements from a commercial fingertip pulse oximeter.

## 3 ∣ Conclusion

A rapid (<3 h) and cost-effective (∼$15/device) fabrication approach was introduced for creating high-performance stretchable electronic circuits with LM interconnects. By replacing sputter-deposited wetting layers with Cu foil coated with LM, this approach eliminates the need for specialized facilities, while enabling access to other diverse and challenging substrates, regardless of their outgassing properties. The Cu-LM film is processed into high-resolution circuit patterns using UV-laser micromachining, a technique used for conventional PCB prototyping. The resulting circuits were able to achieve stable functionality beyond 100% strain and cyclic loading with minimal variations in resistance and capacitance. The practical utility of this approach was demonstrated through a wearable pulse oximeter that conforms to the human finger for the real-time, continuous monitoring of heart rate. The ability to produce complex and stretchable electronic circuits without reliance on cleanroom facilities or specialized fabrication techniques significantly broadens the accessibility of LM-based stretchable electronics, paving the way for advancements in wearable health monitoring, soft robotics, and next-generation bio-integrated devices.

## 4 ∣ Experimental Section

### 4.1 ∣ Cu Foil-LM Circuit Fabrication

Stretchable circuits were manufactured using the fabrication workflow shown in Figure 1D and Video S3. The Cu foil-LM film was prepared by placing Cu foil onto an acrylic substrate and spin-coating a layer of polydimethysiloxane (PDMS; Sylgard 184; Dow; Figure 1D step 1). The PDMS was prepared by combining at oligomer-to-curing agent ratio of 10:1 and vacuum-mixing in a planetary mixer (SpeedMixer DAC 400.2 VAC, FlackTek Inc). After curing in an oven at 80°C for 20 min, the film was peeled off and flipped over to expose the Cu surface while avoiding air pockets (Figure 1D step 2). Next, the Cu surface was spray-coated with 2 passes of eutectic Gallium-Indium (EGaIn) at 50 psi at ambient conditions (Figure 1D step 3) [52]. Circuit traces were created by laser-patterning the EGaIn coated film with a LPKF Protolaser U4 (*λ* = 355 nm, Figure 1D step 4). LM-Cu laser ablation is performed at 3.2 W beam power, 45 kHz, and 400 mm · s^−1^ mark speed, with a s pass at 2.8 W, 45 kHz, and 600 mm · s^−1^ mark speed to remove any residual LM and debris. Appropriate laser settings, minimum trace spacing, and trace width have been determined using power vs. mark speed maps on pure Cu and Cu-LM substrates (Figure S1). To ensure robust contacts between the LM traces and SMD components, all SMD components were wetted with EGaIn in muriatic acid (Sunnyside, 31.45 wt.% HCl) and then placed onto the circuit traces (Figure 1D step 5). Finally, the circuit is encapsulated with PDMS that is then cured in an oven at 60°C for 40 min (Figure 1D step 6). Some circuit components, like sensors, microcontrollers, or batteries, may require longer cure times due to thermal limitations.

### 4.2 ∣ Optical Microscopy

Optical microscopy images were obtained using a Zeiss Axio-Zoom V16 stereo microscope.

### 4.3 ∣ SEM and EDS Analysis

SEM imaging and EDS spectroscopy were performed using FEI Helios Nanolab 660 with the EDAX energy-dispersive and electron backscatter diffraction detector mounted on the microscope.

### 4.4 ∣ Electromechanical Characterization

The electromechanical coupling of the sensors was measured under uniaxial tension tests performed on an Instron 5966 dual column universal testing machine, where samples were held by pneumatically controlled grips. Concurrent resistance and capacitance measurements were obtained using a DMM6500 6.5 digital multimeter.

### 4.5 ∣ Optical Pulse Oximeter

The optical pulse oximeter was configured to collect the reflected light at a frequency of 16.7 Hz. The LED pulse width was set to 411 μs and a current of 6.2 mA. Informed consent of the participant was obtained prior to the study.

## Supplementary Material

SI

Video S1

Video S2

Video S3

Video S4

Additional supporting information can be found online in the Supporting Information section.

**Supporting file:** admt70572-sup-0001-SuppMat.pdf

**Supporting file:** admt70572-sup-0005-VideoS1.mp4

**Supporting file:** admt70572-sup-0005-VideoS2.mp4

**Supporting file:** admt70572-sup-0005-VideoS3.mp4

**Supporting file:** admt70572-sup-0005-VideoS4.mp4

## Data Availability

The data that support the findings of this study are available from the corresponding author upon reasonable request.
