# Supplementary material for: Laser Micromachining of Liquid Metal Patterns for Stretchable Electronic Circuits
Source: Adv Mater Technol. Author manuscript; Available in PMC 2026 Jul 29. (PMC13410773; doi:10.1002/admt.202501838)
Supplement: SI [file NIHMS2189306-supplement-SI.pdf]

Supplementary Information

Laser Micromachining of Liquid Metal Patterns for Stretchable Electronic Circuits

Merjen Palvanova[1], Patrick McManigal[2], Grace Fredrickson[1] and Eric J. Markvicka[1,2,3*]

[1]Department of Mechanical & Materials Engineering, Smart Materials and Robotics Laboratory, University of Nebraska-Lincoln, Lincoln, NE 68588, USA

[2]School of Computing, College of Engineering, Smart Materials and Robotics Laboratory, University of Nebraska-Lincoln, Lincoln, NE 68588, USA

[3]Department of Electrical & Computer Engineering, University of Nebraska-Lincoln, Lincoln, NE 68588, USA

*Corresponding author email: eric.markvicka@unl.edu

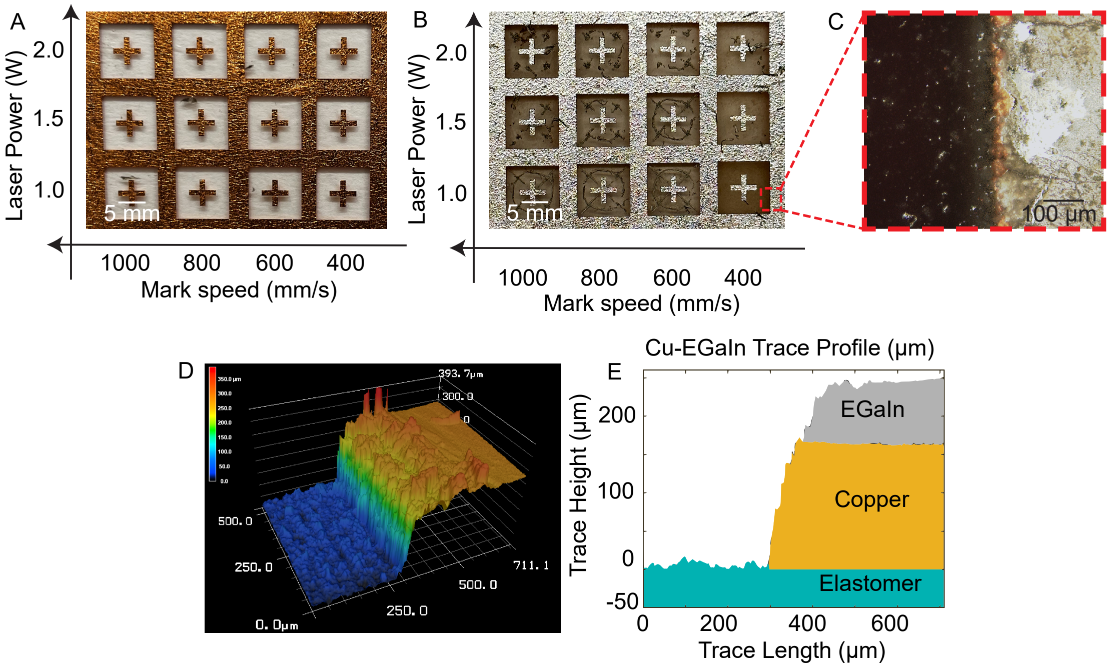

Fig. S1 Optimization of Cu-LM trace patterning. A) Characterization of UV laser settings for pure Cu of laser power versus mark speed map. B) Power versus mark speed map Cu-LM. C) Magnified optical image and D) reconstructed 3D image of Cu-LM trace on the elastomer substrate. E) Laser microscopy measurements of Cu-LM trace profile with elastomer, copper, and EGaIn layers.

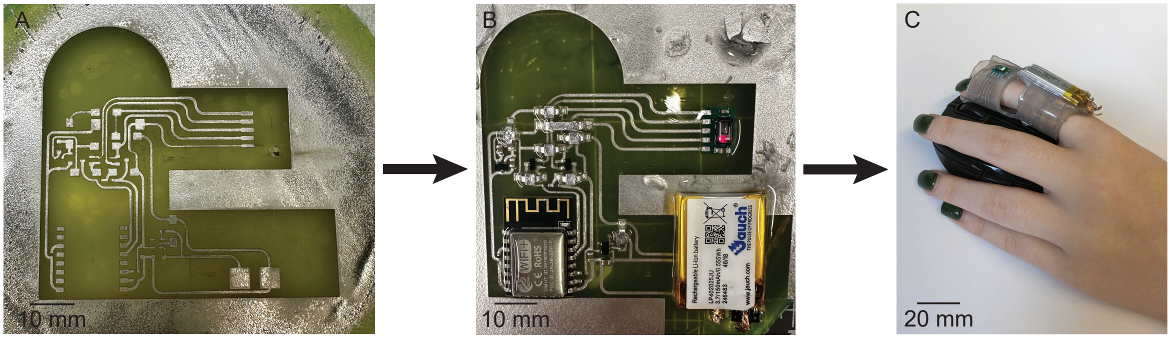

Fig. S2 Pulse-oximeter fabrication. A) UV laser patterned circuit design. B) Circuit components and battery are placed and encapsulated with PDMS. C) The soft matter circuit can be wrapped around the figure to record heart rate.

Table S1: Breakdown of the soft matter LM-Cu circuit board costs.

**Item**	**Quantity**	**Cost**
Cu foil	1 sheet	\$0.20
EGaIn	0.8 grams	\$0.60
Ecoflex 00-30	30 grams	\$1.20
LPKF ProtoLaser U4	30 minutes	\$12.5
	**Total:**	**\$14.50**
